# Unraveling Elastic Fiber-Derived Signaling in Arterial Aging and Related Arterial Diseases

**DOI:** 10.3390/biom15020153

**Published:** 2025-01-21

**Authors:** Mingyi Wang, Kimberly R. McGraw, Robert E. Monticone, Gianfranco Pintus

**Affiliations:** 1 Laboratory of Cardiovascular Science, Intramural Research Program, National Institute on Aging, National Institutes of Health, Baltimore, MD 21224, USA; raginskik@nia.nih.gov (K.R.M.);; 2 Department of Biomedical Sciences, University of Sassari, Viale San Pietro 43/B, 07100 Sassari, Italy; gpintus@uniss.it; 3 Department of Medical Laboratory Sciences, College of Health Sciences, Sharjah Institute for Medical Research, University of Sharjah, Sharjah 27272, United Arab Emirates

**Keywords:** age, elastic fibers, elastic laminae, arterial cells, arterial remodeling, arterial stiffness, arterial disease

## Abstract

Arterial stiffening is a significant risk factor for the development of cardiovascular diseases, including hypertension, atherosclerosis, and arteriopathy. The destruction of elastic fibers, accompanied by vascular inflammatory remodeling, is a key process in the progression of arterial stiffening and related pathologies. In young, healthy arteries, intact elastic fibers create a resilient microenvironment that maintains the quiescence of arterial cells. However, with advancing age, these elastic fibers undergo post-translational modifications, such as oxidation, glycosylation, and calcification, leading to their eventual degeneration. This degeneration results in the release of degraded peptides and the formation of an inflammatory, stiffened niche. Elastic fiber degeneration profoundly impacts the proinflammatory phenotypes and behaviors of various arterial cells, including endothelial cells, smooth muscle cells, macrophages, fibroblasts, and mast cells. Notably, the degraded elastic fibers release elastin-derived peptides (EDPs), which act as potent inflammatory molecules. EDPs activate various arterial cellular processes, including inflammatory secretion, cell migration, proliferation, and calcification, by interacting with the elastin receptor complex (ERC). These elastin-related cellular events are commonly observed with aging and in diseased arteries. These findings suggest that the degeneration of the elastic fiber meshwork is a primary event driving arterial inflammation, stiffening, and adverse remodeling with advancing age. Therefore, preserving elastic fibers and blocking the EDP/ERC signaling pathways may offer promising therapeutic strategies for mitigating age-related arterial remodeling and related arterial diseases.

## 1. Introduction

As we age, the arterial wall progressively stiffens [1,2,3,4]. Arterial stiffness is a major risk factor for the onset and progression of cardiovascular diseases, including hypertension, atherosclerosis, and arteriopathy [4,5,6]. The destruction of elastic fibers (EFs) and the elastic laminae (EL, predominantly composed of contracted EFs), along with vascular inflammatory remodeling processes such as fibro-calcification, are critical tissue events, contributing to arterial stiffening and endothelial dysfunction [1,3,4,5,6,7,8,9,10]. In healthy elastic arteries, intact EFs create an elastic meshwork that not only provides arterial wall elasticity but also creates a quiescent niche for endothelial cells (ECs), vascular smooth muscle cells (SMCs), and fibroblasts (FBs), which may help prevent the onset of inflammation [4,11,12,13].

However, with advancing age, both EFs and EL in the arterial wall undergo post-translational modifications, such as carbamylation, glycosylation, and calcification, leading to their eventual degeneration or fragmentation [4,7,9]. This process and releases elastin-derived peptides (EDPs), which create an inflammatory niche that significantly influences the phenotypes and behaviors of ECs, SMCs, FBs, macrophages, and mast cells [4,7,9,14,15,16,17,18,19]. The degeneration of EFs/EL, along with the release of EDPs, initiates arterial cellular events such as secretion, migration, proliferation, and calcification through the elastin receptor complex (ERC) [14,15,16,17,18,19,20,21,22,23]. These EFs/EL degeneration-associated cellular events are commonly observed in older, diseased, arteries [2,11,24,25,26,27].

These findings suggest that the destruction of the above-mentioned elastic meshwork is a fundamental event underlying arterial inflammation with advancing age. Thus, preserving intact EFs/EL and blocking EDP/ERC signaling may offer promising therapeutic strategies for slowing age-related arterial remodeling and associated diseases.

## 2. Arterial Elastic Fibers/Laminae

### 2.1. Elastic Fibers

**Figure 1:**
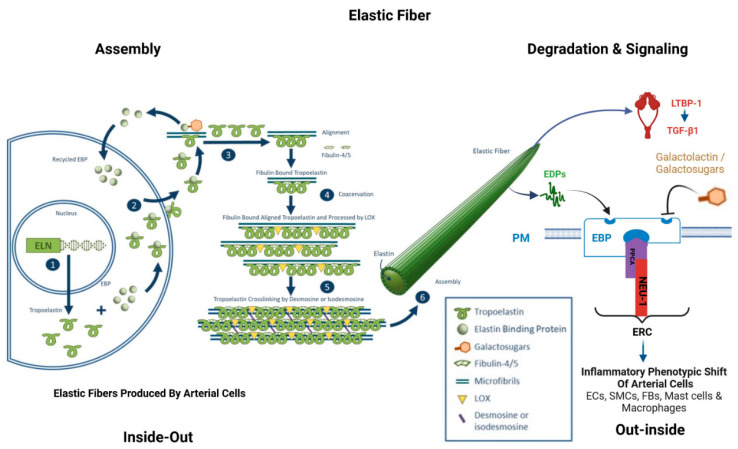
Illustration of assembly and degradation of elastic fibers. Elastic fiber formation and degradation, modified from Kim SH et al. [29] and created with BioRender.com. Notably, inside-out indicates ELN gene expression and translation in the arterial cells and TE secretion and EF assembly in the extracellular space; out-inside indicates EF degeneration, released EDPs and others signaling to the neighboring arterial cells via ERC, EBP = elastin binding protein; ECs = endothelial cells; EDPs = elastin-derived peptides; ELN = elastin gene; ERC = elastin receptor complex; FBs = fibroblasts; SMCs = vascular smooth muscle cells; LTBP-1 = latent TGF binding protein-1; NEU-1 = membrane-bound neuraminidase Neu-1; PPCA = protective protein cathepsin A; PM = plasma membrane; TGF-β1 = transforming growth factor beta1.

The intact EFs structure is a key determinant of the integrity, resilience, and elasticity of arterial walls [1,4,9,12,28]. During the cardiac systolic–diastolic cycle, EFs alternate between stretched and relaxed states approximately 3 billion times over the course of a human lifetime (~70 years). EFs are primarily composed of the protein tropoelastin (TE), encoded by the elastin (ELN) gene, along with its supporting microfibril framework (Figure 1) [5,29,30]. TE, the core protein of EFs, has a half-life of around 70 years, with only 1% of it being renewed per decade [31]. Intact EFs exhibit a very low Young’s modulus (an index of stiffness), ranging from 0.3 to 1.5 MPa, and can be stretched linearly to approximately 1.5 times their original length before tearing [28]. In contrast, collagen fibers have a much higher Young’s modulus, around 1 GPa, making arteries approximately 1000 times stiffer when age-associated collagen fibers replace EFs [32]. As a result, arteries become much stiffer when collagen fibers predominate, as often seen in older arteries, compared to when EFs are dominant in youth.

### 2.2. Elastic Laminae

**Figure 2:**
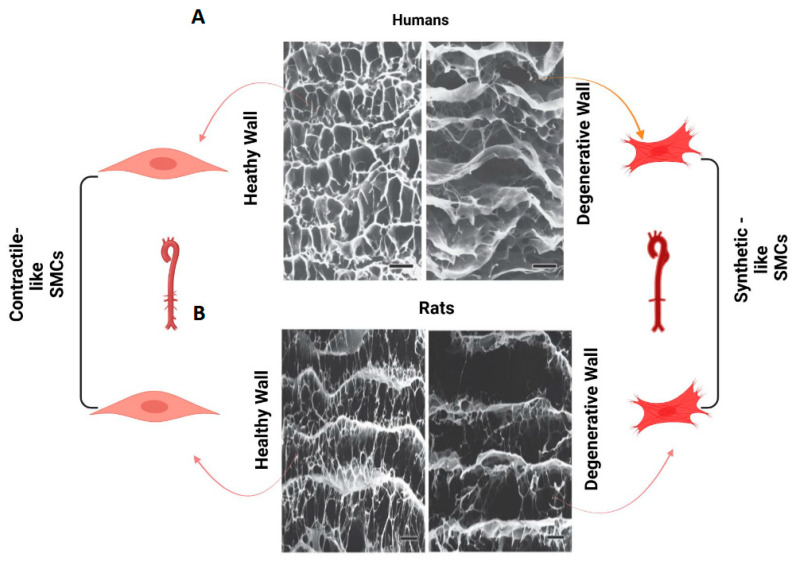
The meshwork of aortic elastic fibers/laminae. Scanning electronic micrograms of normal and degenerated aortic walls in humans (**A**) and rats (**B**), modified from Nakashima Y [12] and created with BioRender.com. Note: It is reasonable to assume that contractile-like SMCs encapsulated in the black enclosure with intact EFs/EL (white) in the normal aortic wall (**left panels**) and synthetic-like cells embedded in destructed EFs/EL (**right panels**) in the degenerated aortic wall in both humans and rats. SMCs = smooth muscle cells.

The EL are the predominant compact microstructure of EFs found in elastic arteries, muscular arteries, and some small resistance arteries. The intact meshwork formed by the EFs/EL imparts elasticity to SMCs, which is crucial for maintaining their structural integrity and functional capacity under healthy normal conditions in both humans and rats (Table 1 and Figure 2, left panels) [1,12,33]. This elasticity may allow arterial SMC to efficiently expand and recoil in response to blood pressure fluctuations during the cardiac cycle, ensuring stable and continuous blood flow throughout the body.

**Figure 3:**
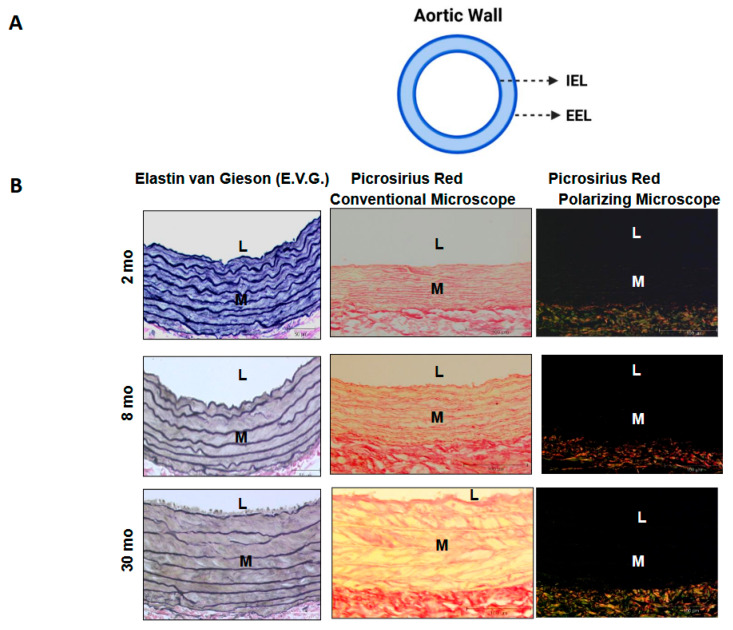
Age-related large arterial remodeling. Illustration of aortic wall (**A**) and the arterial elastic laminae/fibers (dark blue) were stained via Elastic van Gieson (E.V.G.) staining in the large aortic walls of rats, which change with age (from 2 months to 8 months to 3 months) (**left panels**) (**B**), adapted from Wang M et al. [34]. Notably, Picrosirius Red dye collagen stain shows red color under conventional light microscope (**middle panels**), and under a polarizing microscope, type I collagen shows red or yellow, and type III shows green color (**right panels**). IEL = internal elastic lamina; EEL = external elastic lamina.

**Figure 4:**
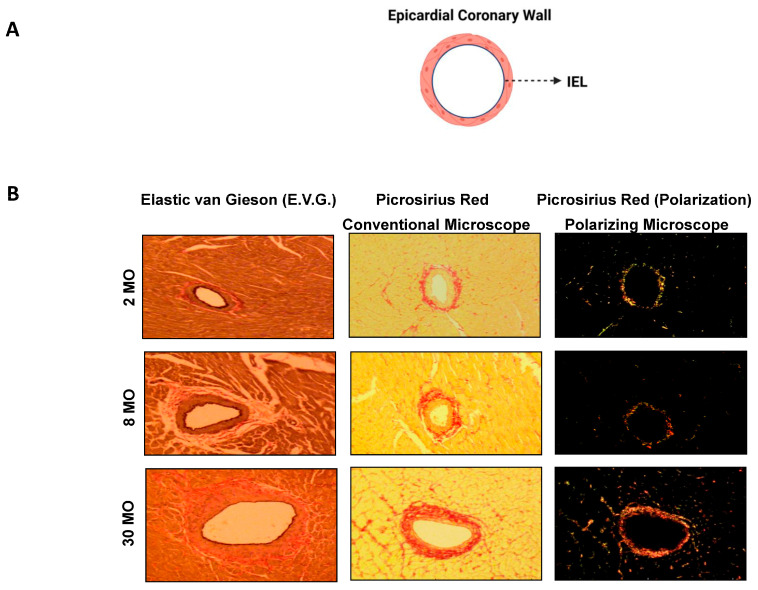
Age-related small arterial remodeling. Illustration of wall (**A**) and epicardial coronary arteries at 2 months, 8 months, and 30 months of age in rats (**B**), adapted from Wang M et al. [35]. Changes in elastic laminae-stained dark blue by Elastic van Gieson; increased collagen stained with Picrosirius Red dye shows red color under conventional light microscope; and under a polarizing microscope, type I collagen shows red or yellow and type III shows green color (**right panels**). IEL = internal elastic lamina.

Two distinct elastic lamina layers are recognized: the internal elastic lamina (IEL), which separates the tunica intima from the tunica media, and the external elastic lamina (EEL), which separates the tunica adventitia from the tunica media in large elastic and muscular arteries (Table 1). Clearly defined IEL, EL, and EEL structures are observed in elastic arteries, such as in 2-month-old rat aorta (Figure 3B, left upper panel), and the IEL is also clearly defined in small muscular arteries, such as epicardial coronary arteries in 2-month-old rats (Figure 4B, left upper panels) [34,35]. In contrast, stiffer collagen fibers (as indicated by the increase in red under a conventional microscope and green or yellow and red fibers under a polarizing microscope) begin to accumulate, seemingly replacing the lost elastic fibers in 30-month-old rat aortae and epicardial coronary artery (Figure 3 and Figure 4, middle and right lower panels) [34,35].

Arteries are categorized into two types: elastic arteries and muscular arteries, which differ primarily in the composition of the tunica media. The tunica media of elastic arteries consists predominantly of EFs/EL, while that of muscular arteries is mainly composed of SMC. Muscular arteries may contain only two defined elastic layers within the tunica media: the IEL and EEL, as seen in arteries such as the mesenteric, internal and external carotid, and tibial arteries. The EEL, situated between the collagen fibers/sheets of the adventitia and the muscle layers of the media, is a well-defined structure in elastic and some muscular arteries.

Notably, both the IEL and EEL play crucial roles in preserving the arterial media’s structural integrity and function and preventing arterial diseases [33,36,37,38,39,40,41,42,43,44]. These elastic layers not only provide mechanical, structural, and functional support to the arterial medial wall but also act as protective barriers for arterial cells. By controlling medial SMC exposure to inflammatory molecules originating from both the intimal and the adventitial areas, the intact IEL and EEL may help maintain arterial cellular quiescence and functions [33,36,37,38,39,40,41,42]. This dual role underscores the importance of the IEL and EEL in maintaining normal arterial cell function, promoting arterial health, and potentially preventing arterial diseases.

## 3. Arterial Elastic Fibers/Elastin Laminae with Aging and Disease

### 3.1. Young, Healthy, Arteries

Under young and healthy conditions, intact EFs/EL play critical structural, functional, and physiological roles in the arterial wall (Table 1). These intact EFs/EL create a quiescent microenvironment that is essential for maintaining the anti-inflammatory and anti-thrombotic properties of overlying ECs and preserving the contractile phenotype of enclosing SMCs within the arterial wall [13]. Additionally, the intact EFs/EL (main TE protein) may provide a stable, resilient, and elastic niche that promotes re-reendothelialization and prevents SMC injuries, thereby supporting arterial homeostasis [13,29].

The IEL, primarily generated from both ECs and SMCs, serves a dual function [39]. First, it acts as a buffer or cushion, orienting both ECs and SMCs to withstand the circumferential and longitudinal strains experienced during the cardiac cycle [13,45]. Second, the IEL forms a physical barrier that prevents the migration and invasion of SMCs and monocytes into the intima-media and mediates communication between ECs and SMCs through fenestrations [13,39,40,42,46,47,48,49,50,51,52]. This barrier function is crucial for preventing endothelial dysfunction, inflammation, intimal–medial thickening, and medial weakening.

The EEL, primarily produced by SMCs, along with other EL, likely plays a key role in limiting medial expansion, bearing longitudinal stress, and restricting inflammation [37,38,39,41,53]. Along with other EL, the EEL organizes arterial components and may prevents excessive arterial wall expansion during the systolic phase of the cardiac cycle [44,54,55]. Additionally, the EEL may prevent the migration and invasion of adventitial fibroblasts, thereby counteracting both medial and intimal thickening [36,44,54,56,57].

Furthermore, the EEL may act as a physical barrier that confines arterial FBs to the adventitia, where they contribute to the production of extracellular matrix (ECM) components and interact with immune cells such as mast cells and macrophages [36,43,44,56,58]. This barrier function may help maintain immune quiescence within the intima and medial wall, preventing the infiltration of immune cells, foam cell formation, and adverse arterial remodeling [38,41,44,58].

### 3.2. Old, Diseased, Arteries

As aging progresses, EFs/EL undergo erosion and even breakdown, with insufficient repair mechanisms in place to restore them (Table 1, Figure 3B and Figure 4B left lower panels) [2,7,29,34,35,54]. The degradation of the elastic meshwork leads to a loss in elasticity and resilience, resulting in the generation of EDPs and releasing latent transforming growth factor 1 (LTBP-1) and transforming growth factor beta 1 (TGF-β1) (Figure 1). This is a major molecular event that promotes inflammatory remodeling, including cellular phenotypic shifts and extracellular matrix restructuring, such as fibrosis, that ultimately contributes to arterial stiffening [1,2,4,5,6,7,8,9,29].

The degradation of EFs/EL releases inflammatory EDPs, which belong to the matrikine family and are primarily located in the intima and media [21]. The most typical EDP is a hexapeptide repeat, VGVAPG. Notably, EDPs are potent pro-inflammatory ligands that interact with ECs and SMCs as well as other vascular inflammatory cells by binding to the ECR (Figure 1), an unusual cell surface receptor [29]. The ECR is a heterotrimeric structure composed of an elastin-binding protein, the membrane-associated protective protein cathepsin A (PPCA), and the membrane-bound neuraminidase Neu-1 (NEU-1) (Figure 1). Notably, ERC activation can be inhibited by galactolatin/galactosugars via induction of EDPs release and dissociation of this complex. Other potential EDP receptors include integrin αvβ3 and galectin-3 in various cell types [20,59].

**Figure 5:**
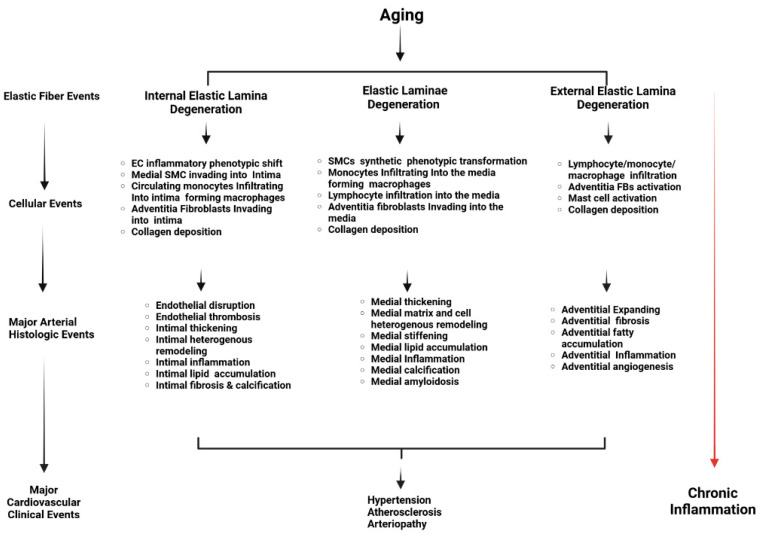
Cellular, histological, and clinical events derived from EFs/EL degeneration in the arterial wall with aging. The schematic representation illustrates the pathophysiological processes involved in arterial aging. It highlights the transition from EFs/EL degeneration events to chronic inflammation through various cellular and histologic changes within the arterial wall. Key components include EL degeneration, endothelial and smooth muscle inflammatory phenotypic transformation, monocytes infiltrating into macrophages, fibroblast activation, and major arterial histologic events. These changes contribute to chronic inflammation and cardiovascular clinical events. This illustration is created with BioRender.com. ECs = endothelial cells; SMCs = vascular smooth muscle cells.

Upon activation by EDPs, the ECR on the plasma membrane of vascular cells triggers alterations in cellular phenotypes, leading to inflammatory changes such as increased migration, invasion, proliferation, and calcification of SMCs (Table 2 and Figure 5). Of note, these phenotypic shifts, along with EFs/EL degeneration, facilitate the onset and progression of cardiovascular diseases, including hypertension, atherosclerosis, and arteriopathy [8,14,16,23,29,46]. Additionally, post-translational modifications, such as the formation of advanced glycation end products, oxidation, calcification, and renin–angiotensin system activation, further increase inflammation, stiffening and weaking of arterial wall [23,29,45,60,61,62].

## 4. Effect of Age-Associated Elastic Degeneration on Phenotypic Shifts of Arterial Cells

Aging or disease accelerates the destruction of EFs/EL. As aging progresses, the arterial wall’s intima and media thicken, and the adventitia expands, leading to an increased presence of EDPs in these regions [4,21,63]. The degradation of EFs/EL and the resulting EDPs serve as potent signaling molecules, driving the inflammatory response in various vascular cells, including ECs, SMCs, FBs, macrophages, and mast cells (Table 2). This process contributes to the initiation and progression of age-related vascular remodeling and associated diseases (Table 3 and Figure 5).

### 4.1. Endothelial Cells

Under normal conditions, arterial ECs are positioned over the IEL via a basement membrane. ECs interact with the IEL, covering its fenestrae, and communicating with medial SMCs likely through direct contact, gap junctions, or extracellular vesicles [13,40,52,64]. These cellular interactions are crucial for maintaining the physiological functions of the endothelium, such as reendothelialization, anti-vasoconstriction, and anti-inflammation, as well as for regulating the contractile state of medial SMCs. This coordination ensures normal vascular tone and blood pressure, both of which are essential for vascular homeostasis and health [13,40,52,64].

Conversely, damage to the EFs/EL and the accompanying release of EDPs generate inflammatory signals [14,16,23]. In human aortic EC, treatment with EDPs triggers the release of proteinases, such as matrix metalloproteinase-1 (MMP-1) [18]. Critically, damage to elastin compromises the endothelium’s ability to release nitric oxide, a gas molecule that plays a key role in promoting SMC relaxation and exerting anti-inflammatory effects, thereby significantly impacting endothelial function [65]. Additionally, EDPs promote the oxidation of low-density lipoproteins (LDLs), enhance monocyte adhesion to the endothelium, and contribute to the development of atherosclerosis [15,24].

Notably, an intact elastin microstructure is essential for EC adhesion, spreading, and cell cycle entry and for ECs communication with SMCs, which are vital for the active repair of endothelial damage and impact of blood flow throughout life [13,48].

### 4.2. Vascular Smooth Muscle Cells

**Figure 6:**
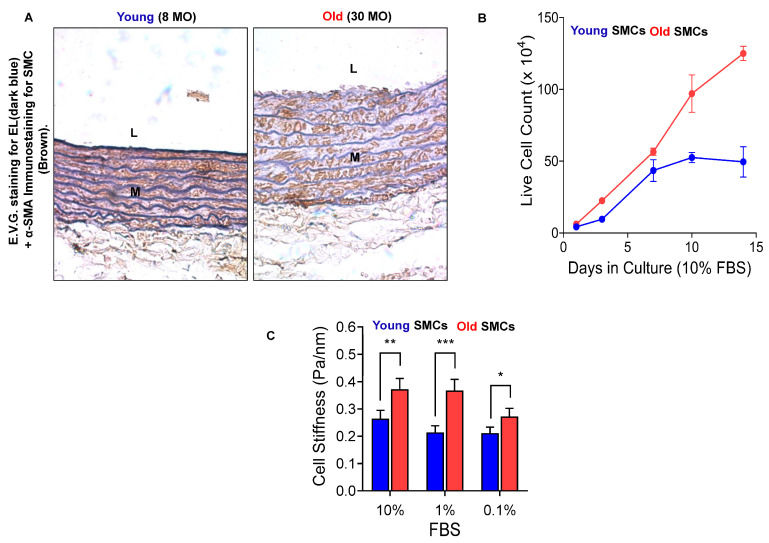
Aging SMCs in the aortic walls. (**A**) Young (left panel) and old (right panel) rat aortic walls stained with E.V.G. for EFs/EL (dark blue) plus immunostaining α-SMA (a SMC marker, brown color) modified from Zhu W et al. [27]. (**B**) Proliferative capacity increases in early passage SMCs isolated from old vs. young FXBN aortic walls. (**C**) Increased stiffness is observed when old vs. young SMCs are compared. L = lumen; M = medium. SMCs = smooth muscle cells; FBS = fetal bovine serum. * *p* < 0.05; ** *p* < 0.01; *** *p* < 0.001.

Aging alters the phenotype of SMCs, characterized by a decline in α-smooth muscle actin (α-SMA) and an increase in cellular stiffness and EL degeneration in the arterial wall, which leads to a higher proliferative capacity in older versus younger SMCs determined by in vivo immunochemistry plus histochemistry (Figure 6A) and in vitro proliferative assay (Figure 6B) and atomic force microscope (ATM) (Figure 6C). The degeneration of EFs/EL, the release of EDPs, or elastin deficiency significantly influence SMC behavior, including inflammatory responses, extracellular matrix secretion, proliferation, migration, and invasion [19,22,30,39,40,42,46,49,50].

The migration and invasion of media SMCs into the intima through the enlarged fenestrae of the IE is a primarily events to the intimal medial thickening and neointima formation [40]. The altered IEL structure is crucial in facilitating the migration and proliferation of medial SMC into the intima, ultimately leading to arterial stenosis [40]. Additionally, EFs/EL degeneration or the released EDPs induce SMC trans-differentiation into osteo-chondrogenic cells, marked by increased MMP-2 and TGF-β1 activation, runt-related transcription factor 2 (RUNX2), and alkaline phosphatase (ALP) while decreased matrix Gla protein (MGP), which contribute to calcification and elastin biomineralization [19,62,66].

### 4.3. Fibroblasts

Arterial FBs are a predominant cell type in the tunica adventitia [67]. Adventitial FBs are activated in responding to the degeneration of EFs/EL, contributing to the production of senescence-associated secretory phenotype, oxidative stress, inflammation, collagen deposition, and intimal medial thickening within the arterial wall [11,36,56,57,68]. Activated FBs become myofibroblasts, which migrate to the media and intima, promoting the intimal medial thickening, inflammation, fibrosis, and calcification [36,57,69,70,71]. Thus, adventitial FBs play a significant role in age-associated adverse cellular and molecular arterial remodeling [2,11,68].

### 4.4. Mast Cells

Mast cells are a type of immune cell predominantly found in the arterial adventitia and thickened intima [43,72,73]. Mast cells are activated by various stimuli, including an age-associated micro-environment [43,72,73]. Upon activation, mast cells release granules containing these inflammatory molecules such as chymase, MMP-2 and MMP-9, monocyte chemoattractant protein1(MCP-1), and C-C chemokine receptor 2 (CCR2) [43,73]. These molecules likely further contribute to the degradation of EFs/EL, exacerbating arterial damage and remodeling [43,72,73]. Notably, mast cells have been observed to infiltrate walls through fragmented Els/EL via CCR2 [74].

### 4.5. Macrophages

Macrophages are immune cells within the arterial wall that differentiate from circulating monocytes and perform various functions, including phagocytosis of pathogens and cellular debris, regulation of inflammation, tissue repair, and modulation of immune responses [75,76,77]. EDPs induce circulating monocytes to adhere to the endothelium, migrate into the subendothelial space, and differentiate into macrophages [15]. These macrophages produce reactive oxygen species (ROSs), which facilitate the oxidation of LDL [15,24].

Resident macrophages are observed in the adventitia of aged arteries [78]. These adventitial macrophages secrete proteinases such as MMP-2, MMP-3, and MMP-12, which may degrade EFs/EL, thus releasing EDPs [58]. The life cycle of macrophages, including their inflammatory responses, chemotaxis, and M1/M2 polarization, can be modulated by EDPs, influencing their behavior and function [14,16,17,55,57]. This process promotes the recruitment of macrophages to sites of injury within the intima, media, or adventitia through the enlarged fenestrations or breaks in the elastic meshwork of arteries [17,58].

The degradation of EFs/EL, the release of EDPs, and the formation of foam cells are critical steps in the initiation and progression of atherosclerosis and arteriopathy [14,16,55]. Foam cells, typically derived from macrophages, are immune cells that have engulfed large amounts of lipids, mainly oxidized LDL [77].

The destruction of EFs/EL likely promote atherosclerosis by inducing phenotypic changes in adventitial macrophages, making them more prone to foam cell formation via vasa vasorum angiogenesis [16,57,76]. While the exact mechanisms linking EFs/EL degeneration to foam cell formation are still under investigation, studies suggest that changes in macrophage behavior—such as increased macrophage infiltration and inflammation within the arterial wall due to elastin degeneration may play a significant role in the processes leading to foam cell formation [14,16,55,57,60,75,76].

## 5. Arterial Diseases Associated with Elastic Fiber Degeneration

The reduced elastin production, fragmented EFs/EL, and loss of elastin are closely associated with several arterial diseases, including hypertension, atherosclerosis, and arteriopathy, such as aneurysms or dissections (Table 3 and Figure 5). Impaired endothelial function and weakened vessels become less resistant to pressure, contributing to chronic blood pressure increase; fragments damage other cells and matrix components, contributing to foam cell aggregation, plaque buildup, and narrowing; and loss of elasticity weakens arterial walls, leading to ballooning and potential rupture. These changes result in arterial stiffening, reducing the ability of arteries to adapt to fluctuations in blood pressure and flow and lowering the threshold for harmful stimuli such as increased pressure and hyperlipidemia. Consequently, the promotion of inflammation is a primary risk factor for several arterial diseases, including hypertension, atherosclerosis, and aneurysms/dissections [4,6,8,12,14,15,41,43,47,55,60,63,74,76,79,80,81,82,83].

### 5.1. Hypertension

Aging significantly increases the incidence of hypertension, particularly advanced hypertension, and is closely linked to the degeneration of arterial elastin or EFs/EL [4,8,34,47,83,84,85,86,87]. In a mouse model of homozygous deficient in elastin (Eln−/−) mice, arterial development is comparable to wild-type (WT) mice until approximately day 17.5 of gestation [85]. Beyond this time point, a marked increase in the number, disorganization, and proliferation of SMC, eventually obstructing the arterial lumen, is observed [85]. Postnatally, the systolic blood pressure (SBP) in Eln−/− mice is double that of WT mice, with a significant increase in arterial stiffness [85]. In addition, heterozygous deficient in elastin (Eln+/−) mice also exhibit hypertension with aging [8,47,84], and Eln+/− mice exhibit higher blood pressure (20–30 mmHg more) than their WT counterparts [46,47,86]. The bioavailability of TGF-β1, modulated by emilin-1, a protein associated with EFs, also influences SBP in mice [5], further suggesting that EFs/EL degeneration may play an important role in the pathogenesis of hypertension.

Notably, an elegant study involving a mouse model expressing human elastin in mice produced a spectrum of elastin expression levels ranging from 30% to 100% of normal [86]. In the mice, the level of elastin was found to be inversely related to arterial stiffness and SBP [84,86]. In addition, in these elastin-deficient mice, vascular stiffening is detectable seven days after birth, but hypertension does not manifest until around the 14th day [5,83]. These findings suggest that changes in mechanical properties may precede changes in SBP and further underscore the pivotal role of elastin in the development of hypertension.

### 5.2. Atherosclerosis

Atherosclerosis is an arterial metabolic disorder closely associated with the degeneration of EFs/EL and can be observed under the microscope [25,88]. The degradation of EFs/EL permits the deep infiltration of lipids and immune cells, such as monocytes, into the aortic wall, leading to the formation of macrophage foam cells and activation of MMP-2 and MMP-9; all of which contribute to or are associated with the formation and disruption of atherosclerotic plaques [25,38,41].

The role of EFs degradation in atherosclerosis has been substantiated by animal models (LDLR−/− and ApoE−/−), particularly through the crossbreeding of mice deficient in MMP-2 and MMP-9 with the atherogenic models [15,89,90]. These findings clearly demonstrate that the degradation of EFs/EL is an element in the development of atherosclerosis.

In LDLR−/− mice fed an atherogenic diet and in obese mice, the degradation of EFs/EL correlates with the formation of atherosclerotic plaques [4,15]. Notably, chronic treatment with EDPs in a mouse model of atherosclerosis has been shown to directly increase the size of atherosclerotic plaques in both LDLR−/− and ApoE−/− mice [15]. Similar effects were observed following the injection of the VGVAPG peptide [15]. Moreover, the absence of phosphoinositide-3-kinase gamma (PI3Kγ) in bone marrow-derived cells prevented EDP-induced atherosclerosis development, demonstrating that PI3Kγ is crucial for EDP-induced arterial lesions [15]. In vitro studies have shown that PI3Kγ is required for EDP-induced monocyte migration and ROS production and that this effect is dependent on NEU-1 activity [15]. Furthermore, the absence of the PPCA-NEU-1 complex in hematopoietic lineage cells abolished the progression of atheroma plaque size and decreased leukocyte infiltration, clearly demonstrating the role of this complex in atherogenesis and suggesting the involvement of endogenous EDPs [15]. This research identifies EDPs as enhancers of atherogenesis and defines the NEU-1/PI3Kγ signaling pathway as a key mediator of this process both in vitro and in vivo, suggesting that these effects are mediated by ERC [15].

### 5.3. Arteriopathy

An aneurysm is a bulge or weakened area in the wall of an artery, which can occur in any artery in the body, such as those in the brain, but is most common in the aorta. Dissection occurs when a tear forms within the aortic wall and causes blood to flow between the laminal layers, thereby creating a false lumen. This can further weaken and expand the artery, significantly increasing the risk of rupture. Notably, dissections most commonly occur in the aorta but can also affect other arteries, such as the coronary arteries. Histologically, both aneurysms and dissections are associated with the degeneration or destruction of EFs/EL, conditions collectively known as elastin-associated arteriopathy [12,14,23,55,58,63,80,81,91].

The EDPs play a significant role in polarizing macrophage-induced inflammation, promoting SMC calcification, and activating matrix metalloproteinases MMP-2/9 and TGF-β1 within the arterial wall [14,19]. These activated MMP-2 and -9 are potent enzymes that effectively cleave EFs both ex vivo and in vivo [92,93]. The incidence of aneurysms, along with EFs degradation, is markedly reduced in genetic models of MMP-2 and MMP-9 knockout (KO) mice compared to WT animals [94]. Furthermore, the use of an antibody targeting the EDP peptide, VGVAPG, significantly reduces aortic MMP-2/-9 activation, EFs/EL fragmentation, macrophage infiltration, and TGF-β1 activity, ultimately mitigating the development of aortic aneurysms in this mouse model [91]. Therefore, preserving the integrity of EFs/EL is crucial in counteracting arterial aneurysms and dissections [55,60,65,80,82,91,94].

## 6. Prevention and Treatment of Elastic Fiber Degeneration

The degradation and cleavage of EFs/EL can be mitigated through both non-pharmaceutical and pharmaceutical approaches [55,60,65,80,82,93]. Pharmaceutical treatments include compounds such as resveratrol and glucagon-like peptides [60,65]. Non-pharmaceutical strategies involve maintaining a healthy diet and regular moderate exercise, which contribute to overall arterial health [80,93]. Additionally, pharmaceutical regimens or gene therapy could be developed to enhance EFs/EL repair, reduce their degradation, and suppress the inflammatory signaling induced by released EDPs [55,60,65,82].

## 7. Conclusions and Future Perspectives

This review emphasizes the critical role that the degeneration of the EFs/EL plays in the progression of arterial aging, which subsequently contributes to the development of various cardiovascular diseases, including hypertension, atherosclerosis, and arteriopathy (Figure 5). The degradation of EFs/EL leads to the release of EDPs, which act as potent signaling molecules driving inflammatory responses and adverse vascular remodeling. These molecular and cellular changes contribute to the stiffening of arteries, loss of elasticity, and increased vulnerability to harmful stimuli, ultimately leading to the onset and progression of age-related arterial diseases.

We believe future research must prioritize developing and refining therapeutic strategies targeting the underlying molecular mechanisms driving EFs/EL degradation and the associated inflammatory responses. A critical direction for investigation lies in the development of pharmacological interventions. Drugs designed to inhibit EDP production, block its receptor ERC signaling, or promote elastin synthesis and repair hold significant promise in addressing the arterial dysfunction caused by elastin degradation [15,29]. Ensuring the efficacy and safety of these compounds in preclinical and clinical settings will be a crucial step toward translating these findings into effective therapeutic options.

We also believe that gene therapies represent a promising avenue for the future, especially in cases where elastin insufficiency is a key factor in the development of arterial diseases. Gene editing technologies employment, including CRISPR/Cas9, may help to correct genetic defects responsible for elastin production impairment, thereby restoring elastin levels in patients [86]. This approach could provide a transformative solution for conditions lacking effective treatments.

We also consider non-pharmaceutical interventions a crucial area worth further exploration. Aspects such as lifestyle modifications, including diet and physical activity, can indeed positively influence vascular health [80,93]. In this regard, a deeper understanding of the molecular mechanisms involved in EFs/EL assembly/disassembly might lead to developing more effective lifestyle recommendations to preserve arterial integrity.

Furthermore, identifying valid biomarkers for EFs/EL degradation and EDP activity would facilitate the early detection of arterial disease, providing novel and better tools for monitoring disease progression and treatment efficacy. In this regard, proteomic/metabolomic and molecular image approaches could uncover valuable insights into the molecular changes associated with elastin degradation.

Finally, although substantial progress has been made in understanding the role of elastin degradation in arterial aging, we believe that further research is essential to precisely uncover the involved molecular and cellular mechanisms. Understanding the interactions between elastin degradation, inflammation, and arterial remodeling is critical for developing targeted therapies addressing the underlying causes of arterial aging and age-associated diseases.

## Figures and Tables

**Table 1:** Aging and the Elastic laminae in the Arterial Wall.

Feature	Young	Old
Elastic laminae (EL)	Abundant, thick, evenly distributed, interwoven with smooth muscle cells	Decrease in number and thickness, fragmentation and calcification, less organization.
Internal Elastic Lamina (IEL)	Sharply defined, continuous, elastic fibers	Fragmented, thickened, and calcified, losing distinct boundary with thickened intima.
External Elastic Lamina (EEL)	Prominent, well-defined, separates media and adventitia	Thinned, fragmented, and calcified, often obscured by adventitial thickening.
Overall effect on arterial wall	Highly elastic, compliant, able to recoil effectively	Stiffened, less compliant, decreased recoil, increased risk of arterial diseases.

**Table 2:** Effects of Elastin Fiber Degeneration on the Phenotype and Behavior of Arterial Cells.

Cell Type	Effects of Elastic Fiber Degeneration and Its Derived Peptides
Endothelial Cells (ECs)	Increase LDL oxidation Increase monocyte adhesion Increase MMP activity Effect on NO production
Smooth Muscle Cells (SMCs)	Stimulate proliferation and migration or invasion Increase vascular tone Increase MMP activity, fibrosis and calcification Impair matrix remodeling
Fibroblasts (FBs)	Stimulate proliferation and migration or invasion Increase collagen deposition and fibrosis Increase senescence, oxidative stress, inflammation
Mast Cells	Trigger degranulation and release inflammatory mediators such as chymase, MMP2/9, and CCR2 etc.
Macrophages	Increase M1/M2 polarization and inflammatory cytokine production Effect on phagocytosis Promote foam cell formation, oxidize stress, and inflammation

**Table 3:** Elastic fiber Degeneration and Arterial disease.

Elastic Fiber Impairment	Arterial Disease	Mechanism
Reduced elastin production	Hypertension	Impair endothelial function, promote fibrosis and weakened vessels become less elasticity, contributing to chronically blood pressure increase.
Fragmented elastic fibers	Arteriosclerosis	Damage fragments and released inflammatory mediators MMP-2/9, contributing to oxidative stress, monocyte migration, and foam cell aggregation, plaque buildup and narrowing.
Loss of elastin	Arteriopathy-Aneurysm/Dissection	Loss of elasticity weakens vessel walls, promoting oxidative stress and inflammatory mediators MMP-2/9 and TGF-β1 activation, eventually leading to ballooning and potential rupture.

## Data Availability

Not applicable.

## References

[1] Berquand A., Wahart A., Henry A., Gorisse L., Maurice P., Blaise S., Romier-Crouzet B., Pietrement C., Bennasroune A., Sartelet H. (2021). Revealing the elasticity of an individual aortic fiber during ageing at nanoscale by in situ atomic force microscopy. Nanoscale.

[2] Fleenor B.S., Marshall K.D., Durrant J.R., Lesniewski L.A., Seals D.R. (2010). Arterial stiffening with ageing is associated with transforming growth factor-β1-related changes in adventitial collagen: Reversal by aerobic exercise. J. Physiol..

[3] Graham H.K., Akhtar R., Kridiotis C., Derby B., Kundu T., Trafford A.W., Sherratt M.J. (2011). Localised micro-mechanical stiffening in the ageing aorta. Mech. Ageing Dev..

[4] Vanalderwiert L., Henry A., Wahart A., Carvajal Berrio D.A., Brauchle E.M., El Kaakour L., Schenke-Layland K., Brinckmann J., Steenbock H., Debelle L. (2024). Metabolic syndrome-associated murine aortic wall stiffening is associated with premature elastic fibers aging. Am. J. Physiol. Cell Physiol..

[5] Cocciolone A.J., Hawes J.Z., Staiculescu M.C., Johnson E.O., Murshed M., Wagenseil J.E. (2018). Elastin, arterial mechanics, and cardiovascular disease. Am. J. Physiol. Heart Circ. Physiol..

[6] Shields K.J., Stolz D., Watkins S.C., Ahearn J.M. (2011). Complement proteins C3 and C4 bind to collagen and elastin in the vascular wall: A potential role in vascular stiffness and atherosclerosis. Clin. Transl. Sci..

[7] Kamenskiy A., Poulson W., Sim S., Reilly A., Luo J., MacTaggart J. (2018). Prevalence of Calcification in Human Femoropopliteal Arteries and its Association with Demographics, Risk Factors, and Arterial Stiffness. Arterioscler. Thromb. Vasc. Biol..

[8] Brengle B.M., Lin M., Roth R.A., Jones K.D., Wagenseil J.E., Mecham R.P., Halabi C.M. (2023). A new mouse model of elastin haploinsufficiency highlights the importance of elastin to vascular development and blood pressure regulation. Matrix Biol..

[9] Doue M., Okwieka A., Berquand A., Gorisse L., Maurice P., Velard F., Terryn C., Molinari M., Duca L., Pietrement C. (2021). Carbamylation of elastic fibers is a molecular substratum of aortic stiffness. Sci. Rep..

[10] Le V.P., Cheng J.K., Kim J., Staiculescu M.C., Ficker S.W., Sheth S.C., Bhayani S.A., Mecham R.P., Yanagisawa H., Wagenseil J.E. (2015). Mechanical factors direct mouse aortic remodelling during early maturation. J. R. Soc. Interface.

[11] Liu X., Jiang D., Huang W., Teng P., Zhang H., Wei C., Cai X., Liang Y. (2021). Sirtuin 6 attenuates angiotensin II-induced vascular adventitial aging in rat aortae by suppressing the NF-κB pathway. Hypertens. Res..

[12] Nakashima Y. (2010). Pathogenesis of aortic dissection: Elastic fiber abnormalities and aortic medial weakness. Ann. Vasc. Dis..

[13] Wilson B.D., Gibson C.C., Sorensen L.K., Guilhermier M.Y., Clinger M., Kelley L.L., Shiu Y.T., Li D.Y. (2011). Novel approach for endothelializing vascular devices: Understanding and exploiting elastin-endothelial interactions. Ann. Biomed. Eng..

[14] Dale M.A., Xiong W., Carson J.S., Suh M.K., Karpisek A.D., Meisinger T.M., Casale G.P., Baxter B.T. (2016). Elastin-Derived Peptides Promote Abdominal Aortic Aneurysm Formation by Modulating M1/M2 Macrophage Polarization. J. Immunol..

[15] Gayral S., Garnotel R., Castaing-Berthou A., Blaise S., Fougerat A., Berge E., Montheil A., Malet N., Wymann M.P., Maurice P. (2014). Elastin-derived peptides potentiate atherosclerosis through the immune Neu1-PI3Kgamma pathway. Cardiovasc. Res..

[16] Kawecki C., Bocquet O., Schmelzer C.E.H., Heinz A., Ihling C., Wahart A., Romier B., Bennasroune A., Blaise S., Terryn C. (2019). Identification of CD36 as a new interaction partner of membrane NEU1: Potential implication in the pro-atherogenic effects of the elastin receptor complex. Cell Mol. Life Sci..

[17] Maeda I., Mizoiri N., Briones M.P., Okamoto K. (2007). Induction of macrophage migration through lactose-insensitive receptor by elastin-derived nonapeptides and their analog. J. Pept. Sci..

[18] Siemianowicz K., Gminski J., Goss M., Francuz T., Likus W., Jurczak T., Garczorz W. (2010). Influence of elastin-derived peptides on metalloprotease production in endothelial cells. Exp. Ther. Med..

[19] Simionescu A., Philips K., Vyavahare N. (2005). Elastin-derived peptides and TGF-beta1 induce osteogenic responses in smooth muscle cells. Biochem. Biophys. Res. Commun..

[20] Pocza P., Süli-Vargha H., Darvas Z., Falus A. (2008). Locally generated VGVAPG and VAPG elastin-derived peptides amplify melanoma invasion via the galectin-3 receptor. Int. J. Cancer.

[21] Maeda I., Kishita S., Yamamoto Y., Arima K., Ideta K., Meng J., Sakata N., Okamoto K. (2007). Immunochemical and immunohistochemical studies on distribution of elastin fibres in human atherosclerotic lesions using a polyclonal antibody to elastin-derived hexapeptide repeat. J. Biochem..

[22] Mochizuki S., Brassart B., Hinek A. (2002). Signaling pathways transduced through the elastin receptor facilitate proliferation of arterial smooth muscle cells. J. Biol. Chem..

[23] Wanga S., Hibender S., Ridwan Y., van Roomen C., Vos M., van der Made I., van Vliet N., Franken R., van Riel L.A., Groenink M. (2017). Aortic microcalcification is associated with elastin fragmentation in Marfan syndrome. J. Pathol..

[24] Fulop T., Larbi A., Fortun A., Robert L., Khalil A. (2005). Elastin peptides induced oxidation of LDL by phagocytic cells. Pathol. Biol..

[25] Van der Donckt C., Van Herck J.L., Schrijvers D.M., Vanhoutte G., Verhoye M., Blockx I., Van Der Linden A., Bauters D., Lijnen H.R., Sluimer J.C. (2015). Elastin fragmentation in atherosclerotic mice leads to intraplaque neovascularization, plaque rupture, myocardial infarction, stroke, and sudden death. Eur. Heart J..

[26] Zarkovic K., Larroque-Cardoso P., Pucelle M., Salvayre R., Waeg G., Negre-Salvayre A., Zarkovic N. (2015). Elastin aging and lipid oxidation products in human aorta. Redox Biol..

[27] Zhu W., Kim B.C., Wang M., Huang J., Isak A., Bexiga N.M., Monticone R., Ha T., Lakatta E.G., An S.S. (2018). TGFβ1 reinforces arterial aging in the vascular smooth muscle cell through a long-range regulation of the cytoskeletal stiffness. Sci. Rep..

[28] Koenders M.M., Yang L., Wismans R.G., van der Werf K.O., Reinhardt D.P., Daamen W., Bennink M.L., Dijkstra P.J., van Kuppevelt T.H., Feijen J. (2009). Microscale mechanical properties of single elastic fibers: The role of fibrillin-microfibrils. Biomaterials.

[29] Kim S.H., Monticone R.E., McGraw K.R., Wang M. (2021). Age-associated proinflammatory elastic fiber remodeling in large arteries. Mech. Ageing Dev..

[30] Li D.Y., Brooke B., Davis E.C., Mecham R.P., Sorensen L.K., Boak B.B., Eichwald E., Keating M.T. (1998). Elastin is an essential determinant of arterial morphogenesis. Nature.

[31] Shapiro S.D., Endicott S.K., Province M.A., Pierce J.A., Campbell E.J. (1991). Marked longevity of human lung parenchymal elastic fibers deduced from prevalence of D-aspartate and nuclear weapons-related radiocarbon. J. Clin. Investig..

[32] Sugita S., Suzumura T., Nakamura A., Tsukiji S., Ujihara Y., Nakamura M. (2021). Second harmonic generation light quantifies the ratio of type III to total (I + III) collagen in a bundle of collagen fiber. Sci. Rep..

[33] Yoshikawa H., Wang S., Seo H., Kurotaki T., Ueki H., Yoshikawa T. (2001). Ultrastructure of aortic elastic fibers in copper-deficient Sika deer (*Cervus nippon* Temminck). J. Vet. Med. Sci..

[34] Wang M., Lakatta E.G. (2002). Altered regulation of matrix metalloproteinase-2 in aortic remodeling during aging. Hypertension.

[35] Wang M., Zhang J., Walker S.J., Dworakowski R., Lakatta E.G., Shah A.M. (2010). Involvement of NADPH oxidase in age-associated cardiac remodeling. J. Mol. Cell. Cardiol..

[36] Han X., Wu A., Wang J., Chang H., Zhao Y., Zhang Y., Mao Y., Lou L., Gao Y., Zhang D. (2018). Activation and Migration of Adventitial Fibroblasts Contributes to Vascular Remodeling. Anat. Rec..

[37] Hill M.A., Nourian Z., Ho I.L., Clifford P.S., Martinez-Lemus L., Meininger G.A. (2016). Small Artery Elastin Distribution and Architecture-Focus on Three-Dimensional Organization. Microcirculation.

[38] Kwon H.M., Kang S., Hong B.K., Kim D., Park H.Y., Shin M.S., Byun K.H. (1999). Ultrastructural changes of the external elastic lamina in experimental hypercholesterolemic porcine coronary arteries. Yonsei Med. J..

[39] Lin C.J., Staiculescu M.C., Hawes J.Z., Cocciolone A.J., Hunkins B.M., Roth R.A., Lin C.Y., Mecham R.P., Wagenseil J.E. (2019). Heterogeneous Cellular Contributions to Elastic Laminae Formation in Arterial Wall Development. Circ. Res..

[40] Lu Y.W., Lowery A.M., Sun L.Y., Singer H.A., Dai G., Adam A.P., Vincent P.A., Schwarz J.J. (2017). Endothelial Myocyte Enhancer Factor 2c Inhibits Migration of Smooth Muscle Cells Through Fenestrations in the Internal Elastic Lamina. Arterioscler. Thromb. Vasc. Biol..

[41] Masuoka T., Hayashi N., Hori E., Kuwayama N., Ohtani O., Endo S. (2010). Distribution of internal elastic lamina and external elastic lamina in the internal carotid artery: Possible relationship with atherosclerosis. Neurol. Med. Chir..

[42] Saito J., Dave J.M., Lau F.D., Greif D.M. (2024). Presenilin-1 in smooth muscle cells facilitates hypermuscularization in elastin aortopathy. iScience.

[43] Tsuruda T., Kato J., Hatakeyama K., Kojima K., Yano M., Yano Y., Nakamura K., Nakamura-Uchiyama F., Matsushima Y., Imamura T. (2008). Adventitial mast cells contribute to pathogenesis in the progression of abdominal aortic aneurysm. Circ. Res..

[44] González-Pérez M., Camasão D.B., Mantovani D., Alonso M., Rodríguez-Cabello J.C. (2021). Biocasting of an elastin-like recombinamer and collagen bi-layered model of the tunica adventitia and external elastic lamina of the vascular wall. Biomater. Sci..

[45] Bell J.S., Adio A.O., Pitt A., Hayman L., Thorn C.E., Shore A.C., Whatmore J.L., Winlove C.P. (2022). Microstructural Characterization of Resistance Artery Remodelling in Diabetes Mellitus. J. Vasc. Res..

[46] Lin C.J., Hunkins B.M., Roth R.A., Lin C.Y., Wagenseil J.E., Mecham R.P. (2021). Vascular Smooth Muscle Cell Subpopulations and Neointimal Formation in Mouse Models of Elastin Insufficiency. Arterioscler. Thromb. Vasc. Biol..

[47] Faury G., Pezet M., Knutsen R.H., Boyle W.A., Heximer S.P., McLean S.E., Minkes R.K., Blumer K.J., Kovacs A., Kelly D.P. (2003). Developmental adaptation of the mouse cardiovascular system to elastin haploinsufficiency. J. Clin. Investig..

[48] Segal S.S. (2015). Integration and Modulation of Intercellular Signaling Underlying Blood Flow Control. J. Vasc. Res..

[49] Smith A.H., Putta P., Driscoll E.C., Chaudhuri P., Birnbaumer L., Rosenbaum M.A., Graham L.M. (2020). Canonical transient receptor potential 6 channel deficiency promotes smooth muscle cells dedifferentiation and increased proliferation after arterial injury. JVS Vasc. Sci..

[50] Sawada H., Katsumata Y., Higashi H., Zhang C., Li Y., Morgan S., Lee L.H., Singh S.A., Chen J.Z., Franklin M.K. (2022). Second Heart Field-Derived Cells Contribute to Angiotensin II-Mediated Ascending Aortopathies. Circulation.

[51] Tada S., Tarbell J.M. (2000). Interstitial flow through the internal elastic lamina affects shear stress on arterial smooth muscle cells. Am. J. Physiol. Heart Circ. Physiol..

[52] Kirby B.S., Bruhl A., Sullivan M.N., Francis M., Dinenno F.A., Earley S. (2013). Robust internal elastic lamina fenestration in skeletal muscle arteries. PLoS ONE.

[53] Clifford P.S., Ella S.R., Stupica A.J., Nourian Z., Li M., Martinez-Lemus L.A., Dora K.A., Yang Y., Davis M.J., Pohl U. (2011). Spatial distribution and mechanical function of elastin in resistance arteries: A role in bearing longitudinal stress. Arterioscler. Thromb. Vasc. Biol..

[54] Dixon A.J., Osei-Owusu P. (2023). Elastin haploinsufficiency accelerates age-related structural and functional changes in the renal microvasculature and impairment of renal hemodynamics in female mice. Front. Physiol..

[55] Dhital S., Rice C.D., Vyavahare N.R. (2021). Reversal of elastase-induced abdominal aortic aneurysm following the delivery of nanoparticle-based pentagalloyl glucose (PGG) is associated with reduced inflammatory and immune markers. Eur. J. Pharmacol..

[56] Kuwabara J.T., Tallquist M.D. (2017). Tracking Adventitial Fibroblast Contribution to Disease: A Review of Current Methods to Identify Resident Fibroblasts. Arterioscler. Thromb. Vasc. Biol..

[57] Maiellaro K., Taylor W.R. (2007). The role of the adventitia in vascular inflammation. Cardiovasc. Res..

[58] Chen Y., Yang X., Kitajima S., Quan L., Wang Y., Zhu M., Liu E., Lai L., Yan H., Fan J. (2022). Macrophage elastase derived from adventitial macrophages modulates aortic remodeling. Front. Cell Dev. Biol..

[59] Patel D., Vandromme S.E., Reid M.E., Taite L.J. (2012). Synergistic activity of alphavbeta3 integrins and the elastin binding protein enhance cell-matrix interactions on bioactive hydrogel surfaces. Biomacromolecules.

[60] Zhao X., Cheng Z., Zhang H., Guo Y., Zhao L., Zhang C., Ye P., Zhang K., Ma X., Wu Q. (2023). Glucagon-Like Peptide-1 Inhibits the Progression of Abdominal Aortic Aneurysm in Mice: The Earlier, the Better. Cardiovasc. Drugs Ther..

[61] Chen J.Z., Sawada H., Ye D., Katsumata Y., Kukida M., Ohno-Urabe S., Moorleghen J.J., Franklin M.K., Howatt D.A., Sheppard M.B. (2021). Deletion of AT1a (Angiotensin II Type 1a) Receptor or Inhibition of Angiotensinogen Synthesis Attenuates Thoracic Aortopathies in Fibrillin1(C1041G/+) Mice. Arterioscler. Thromb. Vasc. Biol..

[62] Parashar A., Gourgas O., Lau K., Li J., Muiznieks L., Sharpe S., Davis E., Cerruti M., Murshed M. (2021). Elastin calcification in in vitro models and its prevention by MGP’s N-terminal peptide. J. Struct. Biol..

[63] Satta J., Laurila A., Paakko P., Haukipuro K., Sormunen R., Parkkila S., Juvonen T. (1998). Chronic inflammation and elastin degradation in abdominal aortic aneurysm disease: An immunohistochemical and electron microscopic study. Eur. J. Vasc. Endovasc. Surg..

[64] Masuda H., Zhuang Y.J., Singh T.M., Kawamura K., Murakami M., Zarins C.K., Glagov S. (1999). Adaptive remodeling of internal elastic lamina and endothelial lining during flow-induced arterial enlargement. Arterioscler. Thromb. Vasc. Biol..

[65] Mieremet A., van der Stoel M., Li S., Coskun E., van Krimpen T., Huveneers S., de Waard V. (2022). Endothelial dysfunction in Marfan syndrome mice is restored by resveratrol. Sci. Rep..

[66] Sinha A., Vyavahare N.R. (2013). High-glucose levels and elastin degradation products accelerate osteogenesis in vascular smooth muscle cells. Diab Vasc. Dis. Res..

[67] Stenmark K.R., Yeager M.E., El Kasmi K.C., Nozik-Grayck E., Gerasimovskaya E.V., Li M., Riddle S.R., Frid M.G. (2013). The adventitia: Essential regulator of vascular wall structure and function. Annu. Rev. Physiol..

[68] Sarad K., Jankowska U., Skupien-Rabian B., Babler A., Kramann R., Dulak J., Jaźwa-Kusior A. (2024). Senescence of endothelial cells promotes phenotypic changes in adventitial fibroblasts: Possible implications for vascular aging. Mol. Cell. Biochem..

[69] Majesky M.W., Dong X.R., Hoglund V., Mahoney W.M., Daum G. (2011). The adventitia: A dynamic interface containing resident progenitor cells. Arterioscler. Thromb. Vasc. Biol..

[70] Wang Y.L., Liu L.Z., He Z.H., Ding K.H., Xue F. (2012). Phenotypic transformation and migration of adventitial cells following angioplasty. Exp. Ther. Med..

[71] Simionescu A., Simionescu D.T., Vyavahare N.R. (2007). Osteogenic responses in fibroblasts activated by elastin degradation products and transforming growth factor-beta1: Role of myofibroblasts in vascular calcification. Am. J. Pathol..

[72] Laine P., Naukkarinen A., Heikkilä L., Penttilä A., Kovanen P.T. (2000). Adventitial mast cells connect with sensory nerve fibers in atherosclerotic coronary arteries. Circulation.

[73] Wang M., Takagi G., Asai K., Resuello R.G., Natividad F.F., Vatner D.E., Vatner S.F., Lakatta E.G. (2003). Aging increases aortic MMP-2 activity and angiotensin II in nonhuman primates. Hypertension.

[74] Zhang J., Chen H., Liu L., Sun J., Shi M.A., Sukhova G.K., Shi G.P. (2012). Chemokine (C-C motif) receptor 2 mediates mast cell migration to abdominal aortic aneurysm lesions in mice. Cardiovasc. Res..

[75] Kim G.D., Ng H.P., Chan E.R., Mahabeleshwar G.H. (2021). Macrophage-Hypoxia-Inducible Factor-1α Signaling in Carotid Artery Stenosis. Am. J. Pathol..

[76] Sedding D.G., Boyle E.C., Demandt J.A.F., Sluimer J.C., Dutzmann J., Haverich A., Bauersachs J. (2018). Vasa Vasorum Angiogenesis: Key Player in the Initiation and Progression of Atherosclerosis and Potential Target for the Treatment of Cardiovascular Disease. Front. Immunol..

[77] Maguire E.M., Pearce S.W.A., Xiao Q. (2019). Foam cell formation: A new target for fighting atherosclerosis and cardiovascular disease. Vascul Pharmacol..

[78] Wang M., Zhao D., Spinetti G., Zhang J., Jiang L.Q., Pintus G., Monticone R., Lakatta E.G. (2006). Matrix metalloproteinase 2 activation of transforming growth factor-beta1 (TGF-beta1) and TGF-beta1-type II receptor signaling within the aged arterial wall. Arterioscler. Thromb. Vasc. Biol..

[79] Long C., Liu H., Zhan W., Chen L., Yu Z., Tian S., Xiang Y., Chen S., Tian X.L. (2022). Chronological attenuation of NPRA/PKG/AMPK signaling promotes vascular aging and elevates blood pressure. Aging Cell.

[80] Aicher B.O., Zhang J., Muratoglu S.C., Galisteo R., Arai A.L., Gray V.L., Lal B.K., Strickland D.K., Ucuzian A.A. (2021). Moderate aerobic exercise prevents matrix degradation and death in a mouse model of aortic dissection and aneurysm. Am. J. Physiol. Heart Circ. Physiol..

[81] Kurihara G., Ujihara Y., Nakamura M., Sugita S. (2023). Delamination Strength and Elastin Interlaminar Fibers Decrease with the Development of Aortic Dissection in Model Rats. Bioengineering.

[82] Guo X., Cai D., Dong K., Li C., Xu Z., Chen S.Y. (2023). DOCK2 Deficiency Attenuates Abdominal Aortic Aneurysm Formation-Brief Report. Arterioscler. Thromb. Vasc. Biol..

[83] Le V.P., Knutsen R.H., Mecham R.P., Wagenseil J.E. (2011). Decreased aortic diameter and compliance precedes blood pressure increases in postnatal development of elastin-insufficient mice. Am. J. Physiol. Heart Circ. Physiol..

[84] Hawes J.Z., Cocciolone A.J., Cui A.H., Griffin D.B., Staiculescu M.C., Mecham R.P., Wagenseil J.E. (2020). Elastin haploinsufficiency in mice has divergent effects on arterial remodeling with aging depending on sex. Am. J. Physiol. Heart Circ. Physiol..

[85] Hirano E., Knutsen R.H., Sugitani H., Ciliberto C.H., Mecham R.P. (2007). Functional rescue of elastin insufficiency in mice by the human elastin gene: Implications for mouse models of human disease. Circ. Res..

[86] Wagenseil J.E., Nerurkar N.L., Knutsen R.H., Okamoto R.J., Li D.Y., Mecham R.P. (2005). Effects of elastin haploinsufficiency on the mechanical behavior of mouse arteries. Am. J. Physiol.-Heart Circ. Physiol..

[87] Li D.Y., Faury G., Taylor D.G., Davis E.C., Boyle W.A., Mecham R.P., Stenzel P., Boak B., Keating M.T. (1998). Novel arterial pathology in mice and humans hemizygous for elastin. J. Clin. Investig..

[88] Yamamoto Y., Sakata N., Meng J., Sakamoto M., Noma A., Maeda I., Okamoto K., Takebayashi S. (2002). Possible involvement of increased glycoxidation and lipid peroxidation of elastin in atherogenesis in haemodialysis patients. Nephrol. Dial. Transplant..

[89] Kuzuya M., Nakamura K., Sasaki T., Cheng X.W., Itohara S., Iguchi A. (2006). Effect of MMP-2 deficiency on atherosclerotic lesion formation in apoE-deficient mice. Arterioscler. Thromb. Vasc. Biol..

[90] Luttun A., Lutgens E., Manderveld A., Maris K., Collen D., Carmeliet P., Moons L. (2004). Loss of Matrix Metalloproteinase-9 or Matrix Metalloproteinase-12 Protects Apolipoprotein E–Deficient Mice Against Atherosclerotic Media Destruction but Differentially Affects Plaque Growth. Circulation.

[91] Guo G., Munoz-Garcia B., Ott C.E., Grunhagen J., Mousa S.A., Pletschacher A., von Kodolitsch Y., Knaus P., Robinson P.N. (2013). Antagonism of GxxPG fragments ameliorates manifestations of aortic disease in Marfan syndrome mice. Hum. Mol. Genet..

[92] Van Doren S.R. (2015). Matrix metalloproteinase interactions with collagen and elastin. Matrix Biol..

[93] Wang M., Zhang L., Zhu W., Zhang J., Kim S.H., Wang Y., Ni L., Telljohann R., Monticone R.E., McGraw K. (2018). Calorie Restriction Curbs Proinflammation That Accompanies Arterial Aging, Preserving a Youthful Phenotype. J. Am. Heart Assoc..

[94] Longo G.M., Xiong W., Greiner T.C., Zhao Y., Fiotti N., Baxter B.T. (2002). Matrix metalloproteinases 2 and 9 work in concert to produce aortic aneurysms. J. Clin. Investig..

